# Integrated Design of Optimized Weighted Deep Feature Fusion Strategies for Skin Lesion Image Classification

**DOI:** 10.3390/cancers14225716

**Published:** 2022-11-21

**Authors:** Niharika Mohanty, Manaswini Pradhan, Annapareddy V. N. Reddy, Sachin Kumar, Ahmed Alkhayyat

**Affiliations:** 1 Department of Information and Communication Technology, Fakir Mohan University, Balasore 756089, India; 2 Department of Information Technology, Lakireddy Bali Reddy College of Engineering, Mylavaram 521230, India; 3 Big Data and Machine Learning Lab, South Ural State University, Chelyabinsk 454080, Russia; 4 College of Technical Engineering, The Islamic University, Najaf 54001, Iraq

**Keywords:** skin lesion classification, feature selection, VGG16, EfficientNet B0, ResNet50, HAM 10000 dataset, BCN 20000 dataset

## Abstract

**Simple Summary:**

The reported global incidences of skin cancer led to the development of automated clinical aids for making proper clinical decision models. Correctly classifying the skin lesions during the early stage may increase the chances of being cured before cancer. However, the skin lesion dataset images pose many critical challenges related to available features to develop classification models with cross-domain adaptability and robustness. This paper made an attempt to select important features from skin lesion datasets for proper skin cancer classification by proposing some feature fusion strategies. Three pre-trained models were utilized to select the important features and then an adaptive weighted mechanism of choosing important features was explored to propose model-based and feature-based optimized feature fusion strategies by optimally and adaptively choosing the weights using a meta-heuristic artificial jellyfish algorithm. The empirical evidence shows that choosing the weights of the pre-trained networks adaptively in an optimized way gives a good starting point for initialization to mitigate the chances of exploding or vanishing gradients.

**Abstract:**

This study mainly focuses on pre-processing the HAM10000 and BCN20000 skin lesion datasets to select important features that will drive for proper skin cancer classification. In this work, three feature fusion strategies have been proposed by utilizing three pre-trained Convolutional Neural Network (CNN) models, namely VGG16, EfficientNet B0, and ResNet50 to select the important features based on the weights of the features and are coined as Adaptive Weighted Feature Set (AWFS). Then, two other strategies, Model-based Optimized Weighted Feature Set (MOWFS) and Feature-based Optimized Weighted Feature Set (FOWFS), are proposed by optimally and adaptively choosing the weights using a meta-heuristic artificial jellyfish (AJS) algorithm. The MOWFS-AJS is a model-specific approach whereas the FOWFS-AJS is a feature-specific approach for optimizing the weights chosen for obtaining optimal feature sets. The performances of those three proposed feature selection strategies are evaluated using Decision Tree (DT), Naïve Bayesian (NB), Multi-Layer Perceptron (MLP), and Support Vector Machine (SVM) classifiers and the performance are measured through accuracy, precision, sensitivity, and F1-score. Additionally, the area under the receiver operating characteristics curves (AUC-ROC) is plotted and it is observed that FOWFS-AJS shows the best accuracy performance based on the SVM with 94.05% and 94.90%, respectively, for HAM 10000 and BCN 20000 datasets. Finally, the experimental results are also analyzed using a non-parametric Friedman statistical test and the computational times are recorded; the results show that, out of those three proposed feature selection strategies, the FOWFS-AJS performs very well because its quick converging nature is inculcated with the help of AJS.

## 1. Introduction

**Figure 1:**
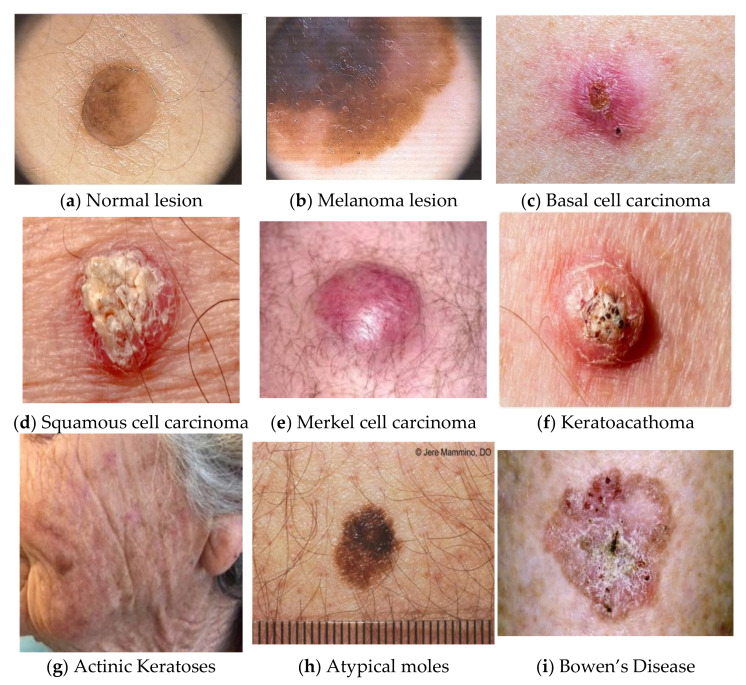
Examples of normal lesions and melanoma in skin lesion images [4]. (**a**) Normal lesion, (**b**) Melanoma lesion, (**c**) Basal cell carcinoma, (**d**) Squamous cell carcinoma, (**e**) Merkel cell carcinoma, (**f**) Keratoacathoma, (**g**) Actinic Keratoses, (**h**) Atypical moles, (**i**) Bowen’s Disease.

Skin lesion mainly refers to a skin area with distinctive characteristics, such as color, shape, size, and texture, from the other surrounding areas of skin. The leading cause of this may be sunburn or contact dermatitis, which causes localized damage to the skin [1,2,3]. The American Society for Dermatologic Surgery describes a skin lesion as an abnormal lump, bump, ulcer, sore, or colored skin area. Other causes of skin lesions or skin patches include any underlying disorder, infections, diabetes, or genetic disorders. It has been seen that this type of skin type may be benign non-harmless or malignant, or premalignant, leading to skin cancer. Freckles or small patches of light brown skin color can be the reason for exposure to the sun. Flat moles are the best examples of skin lesions, and a growing mole with color variation, itching, and bleeding may lead to melanoma lesions, as shown in Figure 1 for regular lesions (Figure 1a) and melanoma lesions (Figure 1b) [4]. 

The study reveals that this skin cancer is the 17th most common cancer worldwide and is a warning phase for researchers and academicians to develop an early detection system for this skin cancer in the form of a computer-based system for effective treatment and better outcomes treatment. The computer-assisted dermoscopic image classification has attracted significant research for its potential to timely and accurately diagnose skin lesions [4,5,6,7]. Scientists, clinicians, analyzers, and experimenters are trying to delve into this area of research to develop models and strategies by exploring artificial intelligence (AI)-, machine learning (ML)-, and deep learning (DL)-based approaches [8,9,10,11]. 

It is evident that DL strategies are being widely used for structure detection by researchers for localization and interpolation of anatomical structures in medical images and to accomplish this task of distinguishing the image features [10,11]. Additionally, the DL methods are highly effective in handling large samples during the training stage, and this network learns valuable representations of the features directly. For example, the convolutional neural network’s (CNN’s) pre-trained architectures can effectively identify and remove the artifacts from the images such as noise. In medial image processing, especially in skin lesion recognition, it is essential to pre-process the image concerning feature selection and feature extraction leading to feature engineering to design an effective and correctly working algorithm [12,13,14,15]. The evolution of transfer learning and its advantages of saving resources with improved efficiency concerning cost and time-consuming issues have widely used CNN’s pre-trained networks in the image analysis research domain [2,11]. In other words, this transfer learning is an ML-based approach where a pre-trained model is reused and customized to develop a new model for a new dataset. For image recognition tasks, the pre-trained models are great because they are easier to use and typically perform better with less training time. It also enables the models to train fast and accurately by extracting the relatively useful features or features of importance at the beginning of training learned from the large datasets [16,17]. The feature level fusion in the classification task has shown improved recognition performance by combining the results of multiple feature selection strategies, thereby identifying a compact set of salient features without losing any data that can improve the recognition accuracy compared to the single base models. Feature fusion, or in other words, the combination of features from different networks, is an omnipresent part of the model learning mechanisms, which is achieved in many ways. The simplest form is the concatenation of outputs of participating networks or using some means or methods of optimizing the weights of the opinions of the participating networks to obtain a good fusion of features having relative discriminative power to design a classification model [18,19,20]. The importance of using optimization in feature fusion is not only to just rank the ranking of features to obtain an optimized version of features, but also the optimized weights help to decide the impact of each feature even if a feature of first rank will have some weighted importance. Being motivated by the advantages of DL-based recognition systems, the use of transfer learning mechanism through CNN’s pre-trained networks, and the feature fusion approach, in this study, we attempted to design a few feature fusion methodologies which call for active fusion approaches resulting to an effective and robust skin lesion classification model. Our prime contributions in this research are: the transfer learning strategy was exploited with the help of CNN’s pre-trained networks for feature selection and feature fusion [2,16,17,18]; the advantages of visual geometry group network (VGG16), EfficientNet B0, and residual neural network (ResNet50) such as low number of parameters and small size filters, multi objective neural architecture optimizing the accuracy and floating point operations with a balanced depth, width, and resolution producing a scalable, accurate and easily deployable model; and the ability to solve the problem of vanishing gradients of those three pre-trained networks have been explored deeply while designing this deep feature fusion model [12,13,14,15]. The key advantages of the ensemble learning mechanism to design a robust feature selection model by proposing combined feature fusion strategies [19,20,21], such as combined feature set (CFS), adaptive weighted feature set (AWFS), model-based optimized weighted feature set (MOWFS), and feature-based optimized weighted feature set (FOWFS), are experimented and validated. In order to reduce the losses and selection of optimized weights of those three pre-trained networks, the advantages of a new meta-heuristic optimizer artificial jellyfish optimizer (AJS) [22,23,24,25,26,27,28,29] was used and finally, the performance of the proposed feature fusion strategies are likened to other combinations of the models with genetic algorithm (GA) [30] and particle swarm optimization (PSO) [31] such as MOWFA-GA, MOWFS-PSO, FOWFS-GA, and FOWPS-PSO, and it was observed that the proposed combination of FOWFS-AJS outperforms the other models used for classification of skin lesion diagnosis.

The rest of the article is organized as follows: the literature on CNN’s pre-trained networks and feature fusion approaches are discussed in Section 2. The pre-trained CNN feature extraction models are discussed in Section 3, the experimentations, results, and discussions are detailed in Section 4. Finally, the conclusion and future scope are given in Section 5.

## 2. Literature Survey

The key challenge associated with the available skin lesion datasets includes the selection of features of importance giving rise to feature selection and/or feature extraction as one the pre-processing task to improve the classification accuracy of the classifiers. This section mainly reviews some related feature selection and feature extraction approaches for image datasets including feature fusion or ensemble techniques. In early studies, the researchers usually used few traditional feature selection methods such as correlation-based feature selection, consistency-based filter, information gain, ReliefF etc., then they shifted their attention to using CNN to extract features. For instance, dense convolutional network (DenseNet), VGG16, Inceptionv3 (GoogLeNet), ResNet, EfficientNet, etc. are the most commonly used pre-trained models for fine-tuning the datasets to improve classification accuracy [2,12,13,14,15].

Lingzhi Kong and Jinyong Cheng [32] proposed classification of COVID-19 X-ray images using DenseNet and VGG models and fine-tuned feature fusion model. First, they applied pre-processing of images and then segmented those images for classification. In addition to this, authors also attempted to resolve the data imbalance problem by introducing fine-tuned global attention block and category attention block to obtain more detailed information of small lesions. Manjary P et al. [33] proposed a classification model to distinguish between natural and computer-generated images by designing a multi-color-space fused EfficientNet using transfer learning methodology which operates in three different color spaces. Ying Guo et al. [34] proposed an EfficientNet based multi view feature fusion model for cervical cancer screening. This proposed model takes the colposcopy images as inputs and tries to extract the features which lead to cervical intraepithelial neoplasia lesions by avoiding the negative effects caused by individual differences and non-cervical intraepithelial neoplasia lesions. An interesting study was carried out by David McNeely-White et al. [35] for comparing the utility of inception and ResNet for as a feature extractor. Authors observed that the features extracted by Inception are very similar to features extracted using ResNet, i.e., the feature set can be very well approximated by an affine transformation of the other. In other words, this literature suggests that for the CNNs, the selection of training set is more important than the selection of pre-trained models.

Yan Wang et al. [36] focused on accurate skin lesion classification by adversarial multimodal fusion with attention mechanism for classification, but before this process, they adopted a discriminator based on adversarial learning to extract the correlated features. This proposed multimodal feature extraction strategy tries to extract the features of the lesion area to enhance the feature vector to obtain more discriminative features. Moreover, the main focus was to consider both correlated and complementary information to design a multimodal fusion strategy. Lina Liu et al. [37] created an automated skin lesion classification model by extracting the region of interest from skin lesson images using ResNet and DenseNet. The authors tried to obtain the mid-level features by studying the relationships among different images based on distance metric learning and gave as an input to the classifiers instead of using the extracted features directly. A study on understanding the efficiency of 17 commonly pre-trained CNN models used for feature extraction was carried out by Samia Benyahia et al. [38]. It has been observed that DenseNet201 along with *k*-nearest neighbor and support sector machine (SVM) outperformed with respect to classification accuracy for the ISIC 2019 dataset. Di Zhuang et al. [39] proposed a cost-sensitive multi-classifier fusion approach for skin lesion image classification by taking the advantage of subjective weights assigned to datasets. That study utilized a concept of cost-sensitive feature by adapting to the different customized cost matrices and twelve different CNN architectures to evaluate the fusion approaches performance.

As per the study, it was seen that the ensemble learning or fusion approach made better predictions and achieved better performance than the single contributing feature or model. The higher predictive accuracy compared to individual models of this ensemble strategy gained wide use in the case of classification. Considering this advantage, many researchers are trying to use this either in the feature level or classifier level. In this section, some works done on this approach are described. Amirreza Mahbod et al. [40] proposed an automatic skin lesion ensemble-based classification model for ISIC 2017 skin lesion classification challenge dataset by combining intra and inter architecture network fusion with multiple sets of CNNs and in that model, the CNNs are pre-trained architectures. Those pre-trained CNNs are able to identify fine-tuned dermoscopic lesion images for the different settings of those models. Similarly, Nils Gessert et al. [41] also proposed an ensemble-based classification model for ISIC 2017 skin lesion classification challenge using EfficientNets, SENet, and ResNeXt WSL. Mohamed A. Elashiri et al. [19] proposed an ensemble-based classification model with the weighted deep concatenated features with long short-term memory. These ensembles of weighted features are basically concatenated features from three CNNs pre-trained models, namely DeepLabv3, ResNet50, and VGG16 integrating the optimal weights of each feature using their proposed hybrid squirrel butterfly search algorithm. Amira S. Ashour et al. [42] also proposed an ensemble-based bag of features strategy for classification of COVID-19 X-ray images. 

Redha Ali et al. [43,44] proposed DL-based skin lesion analysis models in 2019 and 2022. In [43], the authors proposed a CNN-based ensemble method by utilizing VGG19-UNet, DeeplabV3+, and a few other pre-processing methodologies using the ISIC 2018 challenge dataset. Similarly, a DL-based incremental modular network named IMNets was proposed in [44] for medical imaging by using small network modules called as SubNets capable of generating salient features for a particular problem, then larger and more powerful networks were designed by combining these SubNets in different configurations. At each stage, only one new SubNet module underwent learning updates, thereby reducing the computational resource requirements for training in network optimization. Xinzi He et al. [45] proposed a segmentation and classification model by improving the CNNs through a fully transformer network to learn long-range contextual information for skin lesion analysis.

## 3. Methodologies

The preliminary details of VGG16, EfficientNet B0, and ResNet 50 along with their architectures are discussed in this section along with the theory and working process of AJS optimization algorithm. The broad scope of this study along with the proposed deep feature fusion strategies are also detailed along with their workflow diagrams.

### 3.1. CNNs’ Pre-Trained Models for Feature Selection

CNNs’ pre-trained models are saved networks that were previously trained on a large dataset for large-scale image classification and can be used as is or may be customized as per the requirements. This type of architecture of applying the gained knowledge from one source to a different but similar task is widely known as transfer learning. There are many pre-trained models of CNN available and they are being widely used in the field of image processing, such as LeNet, AlexNet, ResNet, GoogleNet or InceptionNet, VGG, DenseNet, EfficientNet, PolyNet, and many more. CNN is basically originated from neural network with convolution layers, pooling layers, activation layers, etc., and those mentioned pre-trained networks are specific CNNs designed for various applications, such as classification and localization [2,12,13,14,15,16,17,31,32,33,34,37].

**Figure 2:**
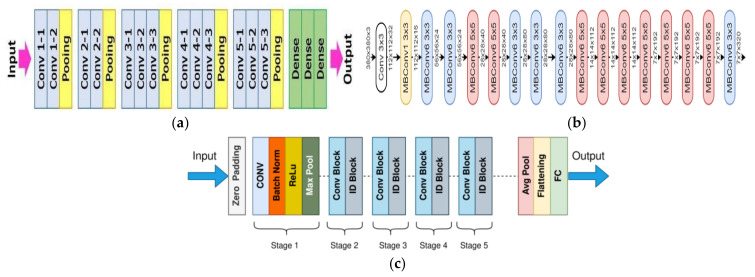
VGG16, EfficientNet B0, and ResNet 50 pre-trained networks architecture. (**a**) VGG16 network architecture [46], (**b**) EfficientNet B0 network architecture [47], (**c**) ResNet50 network architecture [48].

In this work of designing feature fusion strategy for feature selection, three pre-trained CNNs, namely VGG16, EfficientNet B0, and ResNet50 were utilized. The VGG stands for Visual Geometry Group, consisting of blocks composed of 2D convolution and max pooling layers. This has two variants, VGG16 and VGG19, representing 16 and 19 layers in each of them and it has been seen that the performance of VGG16 is equivalent to VGG19; therefore, VGG16 is widely used rather than VGG19. VGG16 was proposed in [46] at the Visual Geometry Lab in Oxford University, United Kingdom in 2014; it is denser with small 3 × 3 filters which provides the effect of a big size filters such as 5 × 5 and 7 × 7, as shown in Figure 2a. The lowering of number of parameters and use of small size filters in the VGG16 network shows the benefit of low computational complexity which gave a new research trend to work with low filters.

EfficientNet uses the neural architecture search to design a new network and it has been scaled up to obtain a family of deep learning models. The EfficientNet B0 was developed using a multi-objective neural architecture optimizing the accuracy and floating point operations. It has been found that this network achieves better accuracy and efficiency in comparison to standard CNN models and taking this EfficientNet B0 as a baseline model, a full family of EfficientNets from EfficientNet B1 to EfficientNet B7 are being developed, and they have shown their accuracy and efficiency on ImageNet. The total number of layers in EfficientNet Bo is 237 and 11 M trainable parameters and the detailed architecture in shown in Figure 2b [47]. This model exacts features throughout the layers by using multiple convolution layers using 3 × 3 receptive field and mobile inverted bottleneck convolution layer. This network employs a balanced depth, width, and resolution which produce a scalable, accurate, and easily deployable model. This EfficientNet was proposed by Mingxing Tan and Quoc V. Le of Google Research in 2019.

Residual network or ResNet is a classic neural network used for many computer vision and image processing tasks and allowed to train more than 150 layers, being the extremely deep neural networks, leading to solving the problem of vanishing gradients introduced by Kaiming He, Xiangyu Zhang, Shaoqing Ren, and Jian Sun in 2015. ResNet50 is a deep network with 5 stages that contains 3 convolutional layers and 1 identity block, which is trained over 23 million parameters and can work very well with 50 neural network layers as shown in Figure 2c [48]. A skip connection is used in the ResNet50 to fetch the earlier parameters to the layers close to the output. It overcomes the vanishing gradient problem.

The concept of wider, deeper, and higher resolution properties of those pre-trained networks giving the network with more filters, more convolution layers and the ability to process the images with larger depth has gained popularity in the field of image processing. Considering those general advantages as well as a few other advantages, such as VGG16 is good at image classification, the effectiveness of model scaling, the proper use of baseline network in EfficientNet B0, and the principle of ResNet50 to build deeper networks and efficiency to obtain number of optimized layers to overcome the vanishing gradient problem, has been the motivation behind this work to design a deep feature fusion strategy for feature selection leading to an effective skin lesion image classification [2,8,9,10,11,12,13,14,15,17,18,19,20].

### 3.2. Artificial Jellyfish Search Algorithm (AJS)

This AJS is one of the newly proposed meta-heuristic swarm-based optimization algorithms derived by simulating the locomotion and dietary patterns [22,23,24,25,26,27,28,29] of jellyfish. Jellyfish are the most efficient swimmers of all aquatic animals widely seen in the oceans having umbrella-shaped bells and trailing tentacles. Their bodies are made up of 98% water which helps them to survive by blending themselves with the direction of current of ocean. The jellyfish swims in the water in such a way that creates two vortex rings, which in turn allows the jellyfish to travel 30% farther on each swimming cycle. From a study, it was observed that jellyfish are excellent swimmers and they utilizes less energy and less oxygen to travel within the water. They have a very simple nervous system which acts as a good receptor to detect light, vibration, and chemicals in the water. They also have a great ability to sense the gravity which allows the jellyfish to traverse in the ocean. The gelatinous skin of this jellyfish helps them to absorb oxygen and their thin hairs help them to bite the food. Jellyfish have stinging cells called nematocysts with tiny needle-like stingers known as tentacles to paralyze the prey before eating. The rising sea temperatures and the dead zones created for other fish or aquatic animals have given a better opportunity to the jellyfish to bloom. 

The jellyfish bloom or flock is being affected by the ecosystem significantly, i.e., the amount of food varies from place to place the jellyfish moves or visits to determine the best place which contains more food. Considering this movement of jellyfish to search for more food in an ocean motivated the design of an AJS based on three idealistic rules:

(a) The movement of the jellyfish is either drawn by the current of the ocean or looking at the swarm and controlling the switching between the mentioned two movements by a time controlled approach;
(b) Being efficient swimmers, jellyfish swim to search for food and try to obtain the locations where a large amount of food is available.

The location simply depends on the quantity of food found and the corresponding objective function of it (i.e., location of jellyfish);

The AJS algorithm basically depends on four ingredients considering the above three rules, namely ocean current; bloom of jellyfish; the time controlled mechanism; and boundary conditions in search spaces and are mathematically formulated and detailed as follows.

(a) Ocean current: The jellyfish is attracted to the large amount of food based on the direction (→) of the current of the ocean and the new location of the jellyfish can be formulated using Equations (1) and (2), respectively.

(1) $$\vec{Ocean_{Current}}=JF^{\#}-\varphi \times rand\left(0,1\right)\times MeanLocation_{JF}$$

(2) $$JF_{i}\left(t+1\right)=JF_{i}\left(t\right)+rand\left(0,1\right)\times \vec{Ocean_{Current}}$$

where, $JF^{\#}$ represents the jellyfish currently at the best location in a swarm or bloom; φ is the distribution coefficient and is >0 related to the direction of $\vec{Ocean_{Current}}$, $JF_{i}$ represents the jellyfish i, and $MeanLocation_{JF}$ represents the new location of each jellyfish.

(b) Jellyfish bloom or swarm: The mobility of the jellyfish is of two types, i.e., passive and active motion, and most jellyfish initially show passive motion during the formation of bloom and they progressively show active motion. Basically, the passive motion of the jellyfish is around their own locations and the corresponding updated location of each jellyfish can be obtained using Equation (3). The $Upper_{bound}$ and $Lower_{bound}$ are the upper and lower bounds of the search space and ω is the length of the movement around the jellyfish’s locations and is called as motion coefficient.

(3) $$JF_{i}\left(t+1\right)=JF_{i}\left(t\right)+\omega \times rand\left(0,1\right)\times \left(Upper_{bound}-Lower_{bound}\right)$$

The active motion can be simulated as

(a) either $JF_{i}$ moves towards $JF_{j}$ or moves away;
(b) $JF_{j}$ other than a $JF_{i}$ is randomly chosen and a vector from $JF_{i}$ to the chosen $JF_{j}$ is used to determine the direction of the movement of jellyfish or motion;
(c) when the food quantity exceeds at the chosen location of $JF_{j}$ that the location of $JF_{i}$, a $JF_{i}$ moves towards a $JF_{j}$;
(d) and if the quantity of the food available to the chosen $JF_{j}$ is lower than that available to a $JF_{i}$, it moves away from it;

This leads every jellyfish to move in a better direction to find food in a bloom and the direction of motion is simulated and the location of the jellyfish is updated using Equations (4) and (5), respectively, where f is an objective function of location of jellyfish.

(4) $$\vec{Motion\;Direction}=\left\{\begin{array}{c} JF_{j}\left(t\right)-JF_{i}\left(t\right)\;\;\;\;\;\;\;\;\;\;\;\;\;\;if\;f\left(JF_{i}\right)\geq f\left(JF_{j}\right)\\ JF_{i}\left(t\right)-JF_{j}\left(t\right)\;\;\;\;\;\;\;\;\;\;\;\;\;\;if\;f\left(JF_{i}\right)<f\left(JF_{j}\right) \end{array}\right.$$

(5) $$JF_{i}\left(t+1\right)=JF_{i}\left(t\right)+rand\left(0,1\right)\times \vec{Motion\;Direction}$$

(c) Time Controlled Mechanism: The passive or active motions of jellyfish in a bloom over a time need to be determined to control the motions of jellyfish towards the ocean current. This time controlled mechanism can be formulated using a time control function $f\left(T_{C}\right)$ which is a random value that changes between (0, 1) over time and a constant c as shown in Equation (6), where maximum number of iterations is given as $Iterations_{max}$ and t is the time specified with respect to the iteration number.

(6) $$f\left(T_{C}\right)=\left|\left(1-\frac{t}{Iterations_{max}}\right)\times \left(2\times rand\left(0,1\right)-1\right)\right|$$

Equation (6) computes the $f\left(T_{C}\right)$ and when this function increases the value of constant c, it signifies that, the jellyfish follow the $\vec{Ocean_{Current}}$ and when $f\left(T_{C}\right)<c$, the jellyfish move inside the bloom. In this case, $f\left(T_{C}\right)=c$ is not known as the time control changes.

(d) Boundary Conditions: The boundary conditions represent the maximum search space defined for the jellyfish. With respect to these boundary conditions (as represented in Equation (7)), when a jellyfish progresses outside the bounds of search area, it will return to the opposite bound. In this equation, $JF_{i,d}$, $JF_{i,d}^{’}$, $Upper_{bound,d}\;$, and $Lower_{bound,d}\;$ represent the location of the $i^{th}$ jellyfish in $d^{th}$ dimension, upper, and lower bounds of the search spaces, respectively.

(7) $$\left\{\begin{array}{c} JF_{i,d}^{’}=\left(JF_{i,d}-Upper_{bound,d}\right)+Lower_{bound}\left(d\right)\;\;\;if\;JF_{i,d}>Upper_{bound,d}\\ JF_{i,d}^{’}=\left(JF_{i,d}-Lower_{bound,d}\right)+Upper_{bound}\left(d\right)\;\;\;if\;JF_{i,d}>Lower_{bound,d} \end{array}\right.$$

### 3.3. Proposed Deep Feature Fusion Approach for Feature Selection

**Figure 3:**
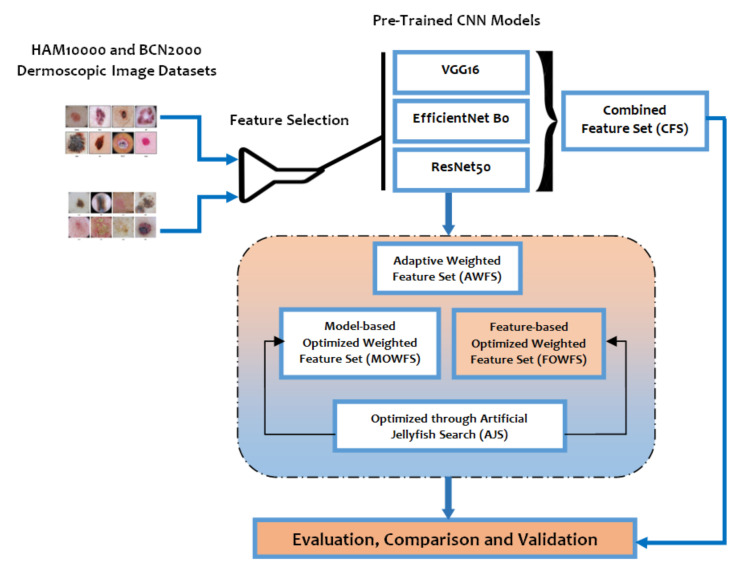
Layout of proposed feature fusion approach for skin lesion data classification.

The broad scope of the proposed deep feature fusion strategy for feature selection of skin lesion classification is outlined in Figure 3. The original feature sets are given as input to the three variants of pre-trained CNN models as an initial phase of experimentation. Considering the contributing factors of ensemble techniques such as (a) the final prediction obtained by combining the results from several base models have achieved better performance and (b) the spread or dispersion of the predictions and model performance are more robust, this study mainly focused on design of ensemble-based feature fusion strategy exploring the deep learning architecture. In this work, four ensemble feature fusion strategies, namely CFS, AWFS, MOWFS, and FOWFS, are proposed, experimented, and validated. 

**Figure 4:**
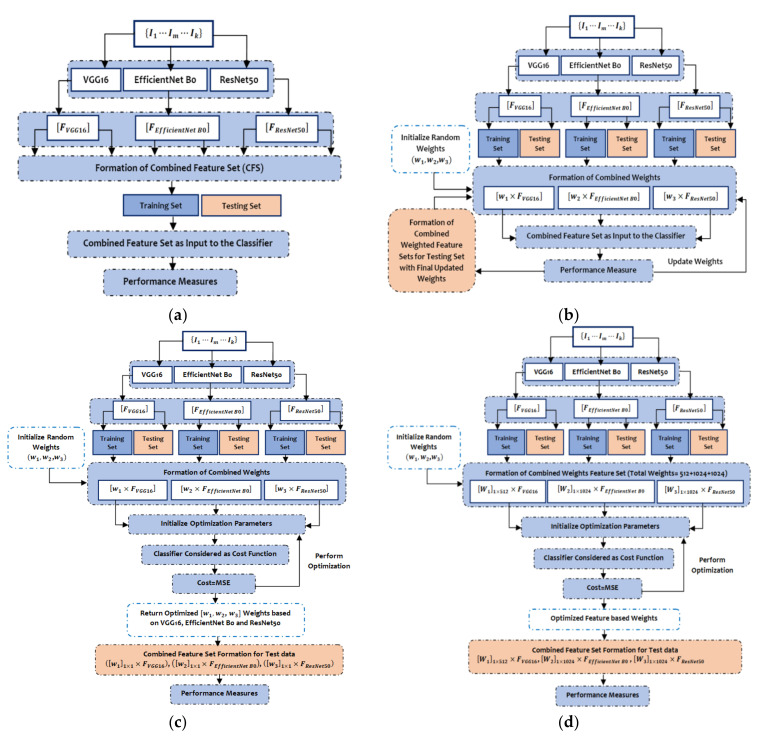
The steps of (**a**) Combined Feature Set (CFS) generation process; (**b**) Adaptive Weighted Feature Set (AWFS) generation process; (**c**) Model-based Optimized Weighted Feature Set (MOWFS) generation process; and (**d**) Feature-based Optimized Weighted Feature Set (FOWFS) generation process.

The predicted features by VGG16, EfficientNet B0, and ResNet50 are 512, 1024, and 1024, respectively, while the input to those three models are images from HAM 10000 [49] and BCN 20000 [50] datasets represented as $\left\{I_{1}\cdots I_{m}\cdots I_{k}\right\}.$ The CFS is one of simplest form of ensemble techniques which simply concatenates the outputs of the three pre-trained models to form a batch of feature set as illustrated in Figure 4a. In the AWFS approach, the weights of those three pre-trained models are initialized to (0, 1) and then the combined feature set is formed by adaptively selecting weights concatenated by the extracted features from the respective pre-trained models, namely $\left[w_{1}\times F_{VGG16}\right],\;\left[w_{2}\times F_{EfficientNet\;B0}\right]$, and $\left[w_{3}\times F_{ResNet50}\right]$ as shown in Figure 4b.

The workflow of the proposed MOWFS is illustrated in Figure 4c, in which initially, the combined feature set is formed same as the AWFS strategy and then, the technique of identifying optimum point considering two special cases (active and passive) motion of AJS optimization algorithm helps to find best cost. In this model-based approach, any one of the classifiers (in our experimentation DT, NB, MLP and SVM) is considered as cost function, where the measured MSE of the opted classification model is taken as the cost and the weights $\left(w_{1},w_{2}\;and\;w_{3}\right)$ are taken as decision variables. This total process is continued for 50 iterations to obtain optimized weights from all three pre-trained models. Then the final ensemble of features is formed for test set as $\left({\left[w_{1}\right]}_{1\times 1}\times F_{VGG16}\right)$, $\left({\left[w_{2}\right]}_{1\times 1}\times F_{EfficientNet\;B0}\right)$, $\left({\left[w_{3}\right]}_{1\times 1}\times F_{ResNet50}\right)$. The process of FOWFS strategy focuses on feature-based optimization of adaptively chosen weights for formation of combined weighted feature set, such as ${\left[w_{1}\right]}_{1\times 512}\times F_{VGG16}$*,* ${\left[w_{2}\right]}_{1\times 1024}\times F_{EfficientNet\;B0}$*,* and ${\left[w_{3}\right]}_{1\times 1024}\times F_{ResNet50}$ with total weights (512+1024+1024). Then, the process of obtaining optimized weights is performed the same as the MOWFS strategy and finally it returns 512 + 1024 + 1024 number of optimized weights based on each feature and the combined feature set is formed as ${\left[w_{1}\right]}_{1\times 512}\times F_{VGG16}$*,* ${\left[w_{2}\right]}_{1\times 1024}\times F_{EfficientNet\;B0}$*,* ${\left[w_{3}\right]}_{1\times 1024}\times F_{ResNet50}$. The total process of this strategy is detailed in Figure 4d. Then, features having weights more than 0.5 are considered as best performing features and are considered for final classification. Finally, the performance of the proposed deep feature fusion strategies such as CFS, AWFS, MOWFS, and FOWFS are evaluated based on each classification model and the proposed optimized strategies are compared with GA and PSO two widely used meta-heuristic optimization techniques though accuracy, precision, sensitivity, and F1-score.

## 4. Experiments, Results, and Discussion

This segment focuses on the experimental stages in order to effectively illustrate the study’s findings. Broadly, the section discusses the datasets and parameter descriptions, the algorithm of the proposed FOWFS feature fusion approach. The experimentation was performed using Intel(R) Core(TM) i5-7200U CPU @ 2.50G Hz with 2.71 GHz processor, 4.00 GB (3.88 GB usable) RAM, 64-bit operating system, x64-based processor operating system, and executed on the platform Google Colab.

### 4.1. Datasets Description

**Figure 5:**
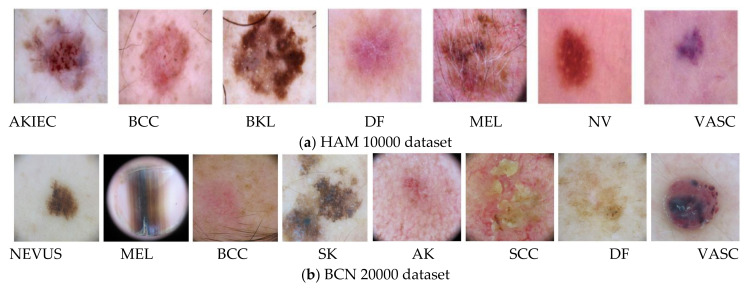
The skin lesions of (**a**) HAM 10000 and (**b**) BCN 20000 datasets.

This study of feature selection and classification was performed on two skin lesion datasets, HAM 10000 and BCN 20000, collected from [49,50]. The HAM 10000 dataset is the abbreviated form of Human Against Machine and it has 10,000 training images for detection of pigmented skin lesions with seven classes. The BCN 20000 dataset is composed of 19,424 demoscopic images of skin lesion collected from a hospital clinic in Barcelona during the period 2010 to 2016 and this dataset has eight classes as detailed in Table 1 and Figure 5a,b.

### 4.2. Parameters Discussion

The various parameters of the network models and optimization techniques used for experimentation of this study and their chosen values are discussed in Table 2.

### 4.3. Algorithm of Proposed FOWFS Feature Fusion Strategy

The working principle of the four feature fusion strategies, CFS, AWFS, MOWFS, and FOWFS, are depicted in Figure 4a–d. The MOWFS and FOWFS strategies are based on optimizing the chosen weights using AJS algorithm. The optimization steps of both are the same, the only difference lies in the formation of combined feature weights as detailed in Section 3.3. The hybridization of AJS for formation of combined feature sets exploring the model-based optimization and each feature-based optimization is depicted in an algorithmic form as given in Algorithm 1.
**Algorithm 1 MOWFS and FOWFS**: Optimized deep feature fusion strategiesFor 100 population $\left(Total_{pop}\right)$ initialize $\;(w_{1},w_{2}\;and\;w_{3})$;

  For $i=1:\;Total_{pop}$
    Calculate MSE using extracted features and cost function of SVM/DT/NB/MLP classifier;
For $\;i=1:\;Total_{pop}$;
    Calculate *time control function* $f\left(T_{C}\right)$ for *t*;
      If $f\left(T_{C}\right)\left(t\right)\geq 0.5$
        Update $w_{1},w_{2}\;\mathrm{and}\;w_{3}$ using Equation (2);
      Else        If $rand\left(0,1\right)>(1-f\left(T_{C}\right)\left(t\right)$        Update $w_{1},w_{2}\;\mathrm{and}\;w_{3}$ using Equation (3);        Else          Update $w_{1},w_{2}\;\mathrm{and}\;w_{3}$ using Equation (5);        End if      End for        End forCheck the boundary conditions such as $Upper_{bound}$ and $Lower_{bound}$, whether $w_{1},w_{2}\;\mathrm{and}\;w_{3}$ range between 0~1;
Choose $w_{1},w_{2}\;\mathrm{and}\;w_{3}$ with minimum MSE;End for: Iterate over 50 iterations;

### 4.4. Result Analysis and Validation

This section discusses the experimental results of all the proposed deep feature fusion approach for skin lesion classification of HAM 10000 and BCN 20000 datasets along with the evaluation and validation of the feature selection strategies. In the first phase of experimentation, the benefit of transfer learning mechanism was achieved for obtaining the better performance with less computational effort. Here, three CNNs’ pre-trained learning models, VGG16, EfficientNet B0, and ResNet50, were used to extract the meaningful features from the new images. 

Table 3 shows the experimental results of those three pre-trained models for both the skin lesion image datasets, which demonstrates the feature acquisition time (in minutes) with respect to the original features. A straightforward comparison was carried out for the accuracy validation with respect to fused feature sets and the highest ranked feature sets (features whose weight>0.5) obtained from three pre-trained models using Decision Tree (DT), Naïve Bayesian (NB), Multi-Layer Perceptron (MLP), and Support Vector Machine (SVM) classifiers as discussed in Table 4, Table 5, Table 6, Table 7, respectively. From those three tables, it can be seen that for both the datasets, initially, the number of features selected from three pre-trained models is 2560 features, which form a fused feature set and the CFS selects 2560 features and as all the features are selected for the classification process, the ranking of features has not been done, therefore there is no improvement in validation accuracy. 

From Table 4, it can be seen that, for the HAM 10000 dataset, the AWFS selects highest ranked feature set with weights of VGG16 (with 512 features) and any one of the other two pre-trained models (with 1024 features) based on DT classifier with an improved accuracy of 94.10%. It can also be inferred that the MOWFS-AJS and FOWFS-AJS have validation accuracy of 94.24% and 94.22%, respectively, with the highest ranked feature set of 1024 and 914 number of features. Considering the improvement in accuracy with respect to CFS, MOWFS-AJS, and FOWFS-AJS, it is clearly evident that with a lower number of feature sets, MOWFS-AJS and FOWFS-AJS achieve 3.14% and 3.12% improved accuracy for HAM 10000 dataset based on DT classifier. Similarly, for the BCN 20000 dataset, the improvement of MOWFS-AJS and FOWFS-AJS over CFS was found to be 7.77% and 7.75%, respectively, with a lower number of features selected as ranked fused feature set based on DT classifier.

The performance based on NB classifier from Table 5 can be detailed as follows. The observed improvements for HAM 10000 dataset of MOWFS-AJS and FOWFS-AJS over CFS were found to be 3.1% and 3.3%, respectively with 1024 + 1024 and 1015 ranked feature sets. Similarly, for the BCN 20000 dataset, the recorded improvements of MOWFS-AJS and FOWFS-AJS over CFS were 6.27% and 6.47%. Additionally, it was seen that the number of features selected for classification by FOWFS-AJS is only 998 features, which is much less in comparison to both strategies.

Table 6 depicts the performance of all proposed feature fusion strategies based on the MLP classifier. From this table, it can be seen that the FOWFS-AJS is outperformed over the rest of the compared methods for both the datasets. The observed improvements for HAM 10000 dataset of MOWFS-AJS and FOWFS-AJS over CFS were found to be 3.3% and 3.58%, respectively, with 512 and 975 features in ranked feature set. Similarly, for the BCN 20000 dataset, the recorded improvements of MOWFS-AJS and FOWFS-AJS over CFS are 5.49% and 5.57% with 512 + 1024 and 929 selected features from the ranked feature set. 

Similarly, the performance based on the SVM classifier for both the datasets are recorded in Table 7. From this table, we can see that the improvements for the HAM 10000 dataset of MOWFS-AJS and FOWFS-AJS over CFS was found to be 3.87% and 5.54%, respectively with 512+1024 and 876 features in the ranked feature set. For the BCN 20000 dataset, the recorded improvements of MOWFS-AJS and FOWFS-AJS over CFS were 4.65% and 6.32% with 512 and 899 selected features from the ranked feature set. From Table 5 to Table 7, the FOWFS-AJS outperformed rest of the proposed feature fusion strategies with respect to validation accuracy measured using NB, MLP, and SVM for both the skin lesion datasets except the performance recorded using DT shows MOWFS-AJS better results in comparison to other strategies (Table 4), but when compared with FOWFS-AJS, it has only 0.02% improved result for both the datasets.

The recognition performance of the three CNNs’ pre-trained models and the proposed strategies, namely CFS, AWFS, MOWFS-GA, MOWFS-PSO, MOWFS-AJS, FOWFS-GA, FOWFS-PSO, and FOWFS-AJS, are recorded in Table 8 and Table 9 for HAM 10000 and BCN 20000 datasets, respectively, by measuring the accuracy, precision, sensitivity, and F1-score based on all four classification algorithms. From both tables, it is observed that the SVM shows better recognition performance and FOWFS-AJS is showing improved recognition rate with respect to all the models considered for comparison.

**Figure 6:**
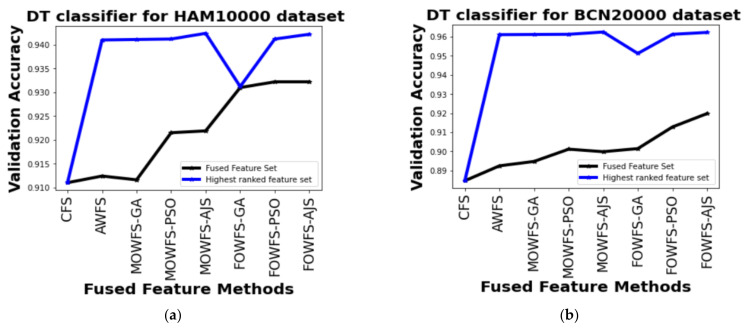
Comparison of validation accuracy for fused feature set and highest ranked feature set using DT classifier for (**a**) HAM 10000 dataset and (**b**) BCN 20000 dataset.

**Figure 7:**
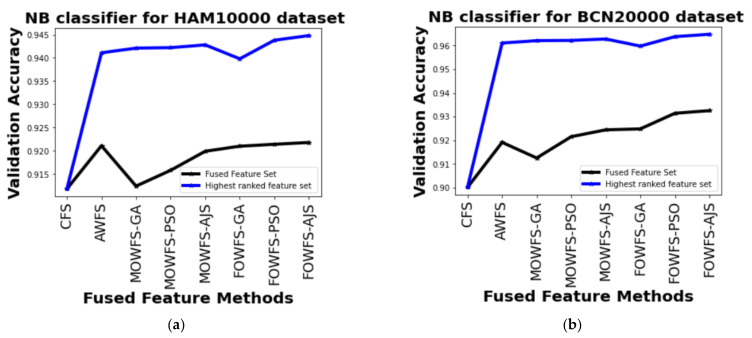
Comparison of validation accuracy for fused feature set and highest ranked feature set using NB classifier for (**a**) HAM 10000 dataset and (**b**) BCN 20000 dataset.

**Figure 8:**
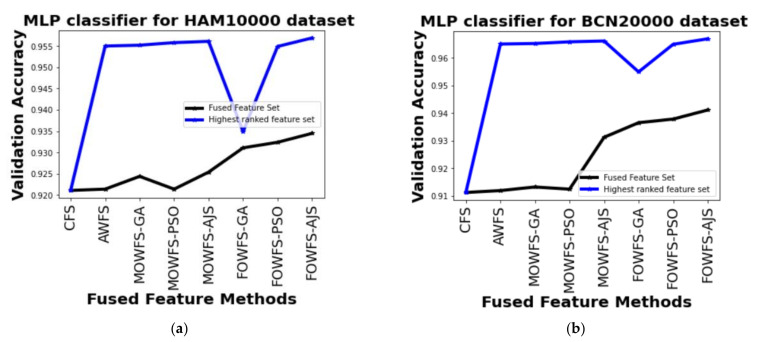
Comparison of validation accuracy for fused feature set and highest ranked feature set using MLP classifier for (**a**) HAM 10000 dataset and (**b**) BCN 20000 dataset.

**Figure 9:**
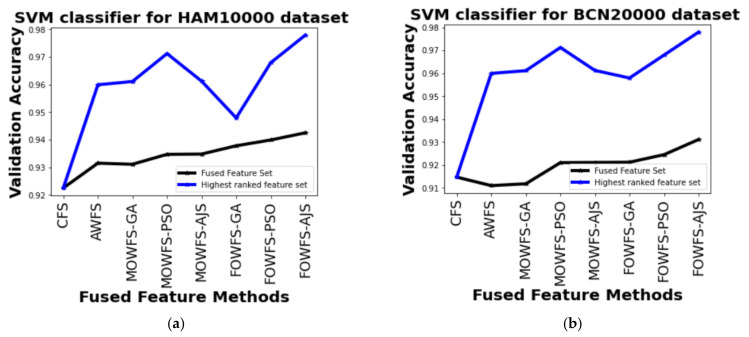
Comparison of validation accuracy for fused feature set and highest ranked feature set using SVM classifier for (**a**) HAM 10000 dataset and (**b**) BCN 20000 dataset.

Further, a straightforward comparison was made considering the observed validation accuracy of all the proposed feature fusion strategies for the combined or fused feature sets and the feature sets obtained after ranking based on all four classifiers for both of the datasets as given in Figure 6, Figure 7, Figure 8, Figure 9. The differences in validation accuracy based on DT classifier for HAM 10000 and BCN 20000 datasets are represented in Figure 6a,b respectively and from this figure, we can see the significant improvement of MOWFS-AJS and FOWFS-AJS over the remaining six strategies and the MOWFS-AJS performed better in this case of classification with 1.09% (fused feature set) and 2.91% (ranked feature set) for HAM 10000 and 3.51% and 7.75% for BCN 20000 datasets. The FOWFS-AJS showed better validation accuracy with respect to the rest of the proposed strategies based on NB, MLP, and SVM classifiers. From Figure 7a,b, it can be seen that FOWFS-AJS over CFS showed improvement of 1% (fused feature set) and 2% (ranked feature set) and 3.24% (fused feature set) and 6.47% (ranked feature set) for HAM 10000 and BCN 20000 datasets, respectively. Similarly, the accuracy recorded based on MLP and SVM classifiers can be summarized as 1.34% (fused feature set),3.51% (ranked feature set), 2% (fused feature set), 5.54% (ranked feature set) for HAM 10000 dataset (Figure 8a and Figure 9a) and 2.99% (fused feature set), 5.57% (ranked feature set) and 1.64% (fused feature set) and 6.35% (ranked feature set) for BNC dataset respectively (Figure 8b and Figure 9b).

**Figure 10:**
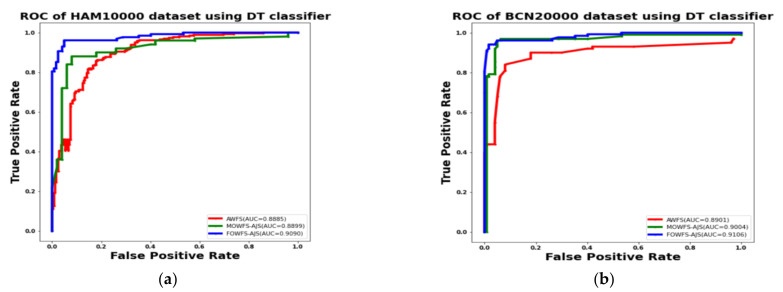
ROC using DT classifier for (**a**) HAM 10000 dataset and (**b**) BCN 20000 dataset.

**Figure 11:**
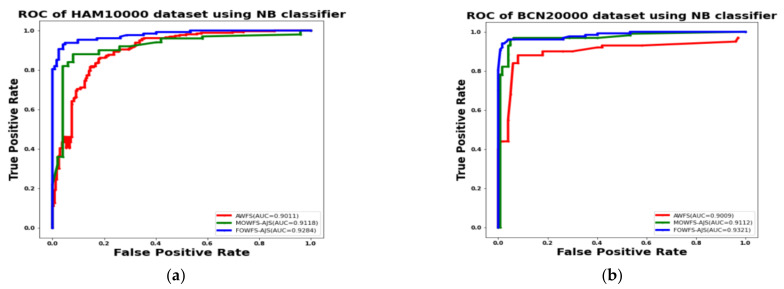
ROC using NB classifier for (**a**) HAM 10000 dataset and (**b**) BCN 20000 dataset.

**Figure 12:**
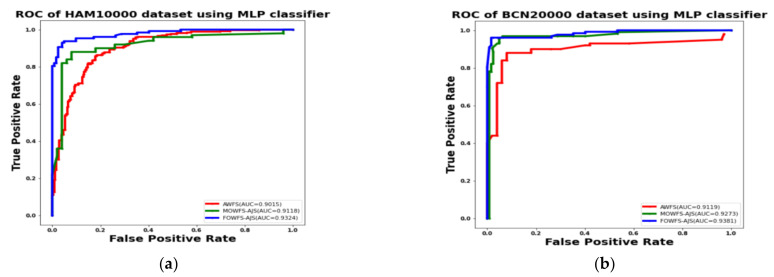
ROC using MLP classifier for (**a**) HAM 10000 dataset and (**b**) BCN 20000 dataset.

**Figure 13:**
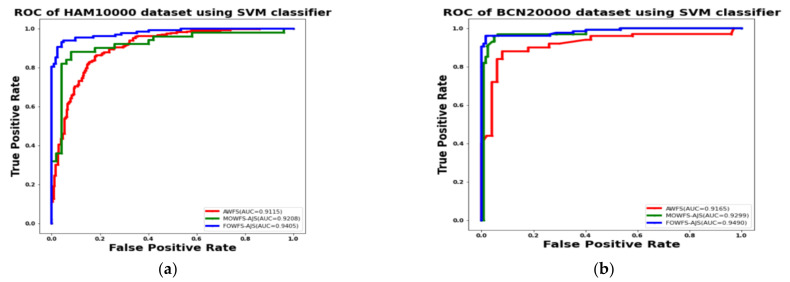
ROC using SVM classifier for (**a**) HAM 10000 dataset and (**b**) BCN 20000 dataset.

Additionally, the area under the receiver operating characteristics curves (AUC-ROC) were plotted to measure the performance and degree of separability amongst the proposed three strategies AWFS, MOWFS-AJS, and FOWFS-AJS to describe how well the models are capable of distinguishing between the classes which are represented in Figure 10, Figure 11, Figure 12, Figure 13 for both datasets based on DT, NB, MLP, and SVM classifiers. From Figure 10a,b, it is observed that FOWFS-AJS showed best accuracy performance with 90.9% and 91.06% for HAM 10000 and BCN 20000 datasets, respectively. Similarly, the recorded performance of the three remaining classifiers can be summarized as: based on NB classifier, the best recorded performance of FOWFS-AJS was 92.84% and 93.21% for HAM 10000 and BCN 20000 datasets, respectively (Figure 11a,b); based on MLP, FOWFS-AJS showed 93.24% and 93.81% for HAM 10000 and BCN 20000 datasets, respectively (Figure 12a,b); and similarly, the SVM recorded a performance of FOWFS-AJS as 94.05% and 94.90%, respectively, for HAM 10000 and BCN 20000 datasets (Figure 13a,b).

**Figure 14:**
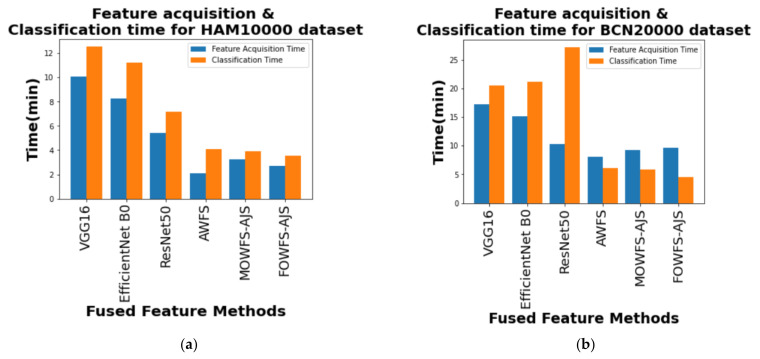
Comparison of mean feature acquisition time vs. classification time of DT, NB, MLP, and SVM classifiers for (**a**) HAM 10000 dataset and (**b**) BCN 20000 dataset.

Finally, a computational comparison (in minutes) was made between the feature acquisition time by the proposed deep feature fusion strategies and the mean of time taken for classification algorithms to classify the skin lesson datasets with the updated feature sets and is shown in Figure 14a,b for HAM 10000 and BCN 20000 datasets, respectively. From those two figures, it is also evident that the proposed FOWFS-AJS comparatively showed better performance with respect to both feature acquisition and classification time for both the datasets.

### 4.5. Validation through Statistical Test

The experimental results were further analyzed using a non-parametric Friedman statistical test [51,52] to determine whether or not there is a statistical difference observed between the models or strategies experimented and compared. Here, this statistical test was utilized to deal with the issue of comparison between all three pre-trained CNNs’ models and the proposed fusion strategies on both HAM 10000 and BCN 20000 datasets. To analyze the performance of VGG16, EfficientNet B0, ResNet50, CFS, AWFS, MOWFS-GA, MOWFS-PSO, MOWFS-AJS, FOWFS-GA, FOWFS-PSO, and FOWFS-AJS, the test was performed from the perspective of average ranking. This Friedman test, which is under the null hypothesis, was computed as follows using Equation (8):

(8) $$\begin{array}{lll} FM_{Stat} & = & \left[\frac{12}{(N\times k\times \left(k+1\right)}\right]\times \sum R^{2}-\left[3\times N\times \left(k+1\right)\right]\\ FM_{Stat} & = & \left[\frac{12}{(N\times k\times \left(3+1\right)}\right]\times \sum 32^{2}+27^{2}+13^{2}-\left[3\times 12\times \left(3+1\right)\right]\\ FM_{Stat} & = & \left[\frac{12}{144}\right]\times \sum \times \left[1024+729+169\right]-144\\ FM_{Stat} & = & \left[0.083\times 1922\right]-144=15.526 \end{array}$$

where $FM_{Stat},\;N,\;k,\;\mathrm{and}\;R$ represent the statistical value, number of datasets, the number of strategies used, and average ranking respectively. The statistical value $FM_{Stat}$ is distributed in line with the Fisherman distribution with $\left(k-1\right)$ and $(\left(k-1)(\right)\left(T-1)\right)$ degrees of freedom. The result of this test is $R=\left[21\;21\;18\;16\;12\;10\;4\;8\;14\;6\;2\right]$ and the calculated $FM_{Stat}=19.7988.$ The critical value is 2.9782 under the significance level α=0.05 for N=2 and k=11; it is evident that the critical value is smaller than the observed values of all $FM_{Stat}$ on all evaluation metrics. This means that the null hypothesis on all evaluations metrics is rejected under this test and the proposed FOWFS-AJS deep feature fusion strategy achieves satisfactory performance on two datasets and eleven compared models.

### 4.6. Discussions on Key Findings

The key findings of this research are as follows. The performance of the transfer learning at the feature level based on the CNNs’ three pre-trained networks achieved optimal performance faster than any other traditional feature selection models and the ensemble learning of features used to design the feature fusion models (for example, CFS) from the output of those three pre-trained networks showed their good performance to design a robust classifier for skin lesion datasets. From the experimentation, it was seen that only designing a CFS model based on basic fusion strategy does not achieve better leverage, therefore the weighted approach of selecting features and forming features sets through AWFS was experimented and shown to have better performance over CFS. Rather than only using feature fusion, it was seen that the strategy for decision on feature fusion approach by utilizing the AJS optimizer to identify the optimum point considering two special cases (active and passive) motions of this algorithm helped to find the best cost. In this study, two decision-based feature fusion models, namely model-based and feature based strategies formed by adaptively choosing the optimal weights such as MOWFS-AJS and FOWFS-AJS have showed their relatively good performance. The MSE of both model-based and feature-based strategies are measured as cost function, where the measured MSE of the opted classification model is taken as the cost and the weights $\left(w_{1},w_{2}\;and\;w_{3}\right)$ are taken as decision variables. This total process was continued for 50 iterations to obtain optimized weights from all three pre-trained models. Thus, the final ensemble of features was formed for test set as $\left({\left[w_{1}\right]}_{1\times 1}\times F_{VGG16}\right)$, $\left({\left[w_{2}\right]}_{1\times 1}\times F_{EfficientNet\;B0}\right)$, $\left({\left[w_{3}\right]}_{1\times 1}\times F_{ResNet50}\right)$ for model-based strategy. The feature-based strategy focused on feature-based optimization of adaptively chosen weights for formation of combined weighted feature set such as ${\left[w_{1}\right]}_{1\times 512}\times F_{VGG16}$*,* ${\left[w_{2}\right]}_{1\times 1024}\times F_{EfficientNet\;B0}$ and ${\left[w_{3}\right]}_{1\times 1024}\times F_{ResNet50}$ with total weights (512 + 1024 + 1024). Then, the process of obtaining optimized weights is performed and finally it returns 512 + 1024 + 1024 optimized weights based on each feature and the combined feature set is formed as ${\left[w_{1}\right]}_{1\times 512}\times F_{VGG16}$*,* ${\left[w_{2}\right]}_{1\times 1024}\times F_{EfficientNet\;B0}$*,* ${\left[w_{3}\right]}_{1\times 1024}\times F_{ResNet50}$. Then, features having weights>0.5 were considered as best performing features and were considered for final classification. The performance of the proposed deep feature fusion strategies was evaluated based on each classification model and the proposed optimized strategies were compared with GA and PSO, two widely used meta-heuristic optimization techniques, through accuracy, precision, sensitivity, and F1-score. Finally, the Friedman statistical test was performed to statistically validate the proposed strategies.

The empirical evidence showed that choosing the weights of the pre-trained networks adaptively in an optimized way gave a good starting point for initialization to mitigating the chances of exploding or vanishing gradients, thus the performance of FOWFS-AJS with SVM learning leveraged the existing network for both the skin lesion datasets and the advantage of properly selecting rich and informative beneficial feature through this feature-based optimized approach received better attention during the experimentation and validation processes.

## 5. Conclusions

Visual inspection and manual examination of skin lesion images has been a burden to the physicians and clinicians to detect melanoma. With the advancements of technology and computational resources, academicians and researchers are trying to develop computational models and AI, ML, and DL have given a new direction to this area of research. In this work, we tried to propose feature level fusion strategies by exploring the DL approaches which in turn help for proper classification. An empirical study was attempted for design of combined, weighted, and optimized strategies of feature selection by exploring the feature fusion approach for classification of skin lesion image classification. The key advantages of transfer learning through the CNNs’ pre-trained networks, fusion approach, selection of features sets by adaptively choosing the weights (model based and feature based) with a new meta-heuristic optimizer AJS was experimented for two skin lesion datasets and then validated through four state-of-the art classifiers, namely DT, NB, MLP, and SVM. The validations of the proposed strategies were performed based on classification accuracies, precision, sensitivity, and F1-score, the difference between the validation accuracies and the AUC-ROC curves were plotted. Extensive comparative studies and the computational time taken for acquisition of features to form features along with statistical validations were performed and the outcome of this empirical research led to conclude that in this experimental setting, the feature sets generated through the proposed FOWFS-AJS leveraged the SVM classifier to classify the HAM 10000 and BCN 20000 skin lesion datasets. This work only explored three pre-trained networks and can be further experimented for few more pre-trained networks to establish the capability of transfer learning. Further, this research can be implemented for other domains of research and specifically, the decision fusion approach can be further explored by utilizing many other upcoming meta-heuristic optimization techniques and a few other skin lesion datasets can also be experimented.

## Figures and Tables

**Table 1:** Datasets and description of skin lesion classes.

Dataset	Classes
HAM 10000	Actinic keratoses and intraepithelial carcinoma/Bowen’s disease (AKIEC), basal cell carcinoma (BCC), benign keratosis-like lesions (BKL), dermatofibroma (DF), melanoma (MEL), melanocytic nevi (NV) and vascular lesions (VASC).
BCN 20000	Nevus, melanoma (MEL), basal cell carcinoma (BCC), seborrheic keratosis (SK), actinic keratosis (AK), squamos cell carcinoma (SCC), dermatofibroma (DF), and vascular lesions (VASC).

**Table 2:** Parameters and their chosen values.

Network Models and Optimization Techniques	Parameters and Their Associated Values
VGG16	16 weight layers
EfficientNet B0	237 weight layers
ResNet50	50 weight layers
AWFS	Total weights:3; $w_{1}$ dimension = 1 × 1; $w_{2\;}$ dimension = 1 × 1; $w_{2\;}$ dimension = 1 × 1
MOWFS	Total weights:3; $w_{1}$ dimension = 1 × 1; $w_{2\;}$ dimension = 1 × 1; $w_{2\;}$ dimension = 1 × 1
FOWFS	Total weights:3; $w_{1}$ dimension = 1 × 504; $w_{2\;}$ dimension = 1 × 1024; $w_{2\;}$ dimension = 1 × 1024
GA	Number of decision variables = 3; Maximum number of iterations = 50; Population size = 10; Selection method-Roulette wheel
PSO	Number of decision variables = 3; Maximum number of iterations = 50; Number of particles = 10; Inertia weight = 1; Inertia weight damping ratio = 0.99; Personal learning coefficient = 1.5; Global learning coefficient = 2.0
AJS	Number of decision variables = 3; Maximum number of iterations = 50; Population size = 10

**Table 3:** Feature acquiring time.

CNN Pre-Trained Models	Datasets	Original No. of Features	Feature Acquisition Time
VGG16	HAM 10000	512	10.11
EfficientNet B0		1024	8.24
ResNet50		1024	5.42
VGG16	BCN 20000	512	17.21
EfficientNet B0		1024	15.11
ResNet50		1024	10.29

**Table 4:** Validation accuracy of fused feature sets vs. ranked feature sets based on DT.

Fused Feature Configurations	Datasets	Dimensionality (Fused Feature Set)	Validation Accuracy (Fused Feature Set)	Dimensionality (Highest Ranked Feature Set)	Validation Accuracy (Highest Ranked Feature Set)
CFS	HAM 10000	2560	0.9110	2560	0.9110
AWFS		2560	0.9124	512+1024	0.9410
MOWFS-GA		2560	0.9116	512	0.9411
MOWFS-PSO		2560	0.9215	512	0.9412
MOWFS-AJS		2560	0.9219	1024	0.9401
FOWFS-GA		2560	0.9310	1015	0.9312
FOWFS-PSO		2560	0.9322	954	0.9412
FOWFS-AJS		2560	0.9322	914	0.9422
CFS	BCN 20000	2560	0.8847	2560	0.8847
AWFS		2560	0.8925	512	0.9610
MOWFS-GA		2560	0.8948	512 + 1024	0.9611
MOWFS-PSO		2560	0.9012	512 + 1024 + 1024	0.9612
MOWFS-AJS		2560	0.8999	1121	0.9602
FOWFS-GA		2560	0.9015	998	0.9512
FOWFS-PSO		2560	0.9128	1019	0.9611
FOWFS-AJS		2560	0.9198	925	0.9622

**Table 5:** Validation accuracy of fused feature sets vs. ranked feature sets based on NB.

Fused Feature Configurations	Datasets	Dimensionality (Fused Feature Set)	Validation Accuracy (Fused Feature Set)	Dimensionality (Highest Ranked Feature Set)	Validation Accuracy (Highest Ranked Feature Set)
CFS	HAM 10000	2560	0.9118	2560	0.9118
AWFS		2560	0.9211	512	0.9411
MOWFS-GA		2560	0.9124	512	0.9421
MOWFS-PSO		2560	0.9158	1024	0.9422
MOWFS-AJS		2560	0.9199	1024 + 1024	0.9428
FOWFS-GA		2560	0.9210	995	0.9391
FOWFS-PSO		2560	0.9214	961	0.9438
FOWFS-AJS		2560	0.9218	1015	0.9448
CFS	BCN 20000	2560	0.9001	2560	0.9001
AWFS		2560	0.9191	512 + 1024	0.9611
MOWFS-GA		2560	0.9125	1024	0.9621
MOWFS-PSO		2560	0.9215	512 + 1024	0.9622
MOWFS-AJS		2560	0.9244	512 + 1024 + 1024	0.9628
FOWFS-GA		2560	0.9248	1115	0.9594
FOWFS-PSO		2560	0.9314	1245	0.9632
FOWFS-AJS		2560	0.9325	998	0.9648

**Table 6:** Validation accuracy of fused feature sets vs. ranked feature sets based on MLP.

Fused Feature Configurations	Datasets	Dimensionality (Fused Feature Set)	Validation Accuracy (Fused Feature Set)	Dimensionality (Highest Ranked Feature Set)	Validation Accuracy (Highest Ranked Feature Set)
CFS	HAM 10000	2560	0.9211	2560	0.9211
AWFS		2560	0.9214	1024	0.9550
MOWFS-GA		2560	0.9244	512 + 1024	0.9552
MOWFS-PSO		2560	0.9214	512 + 1024	0.9558
MOWFS-AJS		2560	0.9254	512	0.9561
FOWFS-GA		2560	0.9311	915	0.9342
FOWFS-PSO		2560	0.9324	898	0.9537
FOWFS-AJS		2560	0.9345	975	0.9562
CFS	BCN 20000	2560	0.9112	2560	0.9112
AWFS		2560	0.9119	512+1024	0.9650
MOWFS-GA		2560	0.9132	1024+1024	0.9652
MOWFS-PSO		2560	0.9124	512	0.9658
MOWFS-AJS		2560	0.9312	512+1024	0.9661
FOWFS-GA		2560	0.9365	1124	0.9549
FOWFS-PSO		2560	0.9378	954	0.9649
FOWFS-AJS		2560	0.9411	929	0.9669

**Table 7:** Validation accuracy of fused feature sets vs. ranked feature sets based on SVM.

Fused Feature Configurations	Datasets	Dimensionality (Fused Feature Set)	Validation Accuracy (Fused Feature Set)	Dimensionality (Highest Ranked Feature Set)	Validation Accuracy (Highest Ranked Feature Set)
CFS	HAM 10000	2560	0.9225	2560	0.9225
AWFS		2560	0.9315	1024 + 1024	0.9599
MOWFS-GA		2560	0.9311	512	0.9611
MOWFS-PSO		2560	0.9347	512 + 1024	0.9712
MOWFS-AJS		2560	0.9348	512 + 1024	0.9612
FOWFS-GA		2560	0.9378	1125	0.9479
FOWFS-PSO		2560	0.9399	897	0.9679
FOWFS-AJS		2560	0.9425	867	0.9779
CFS	BCN 20000	2560	0.9147	2560	0.9147
AWFS		2560	0.9110	1024	0.9599
MOWFS-GA		2560	0.9118	1024 + 512	0.9611
MOWFS-PSO		2560	0.9210	1024 + 1024	0.9712
MOWFS-AJS		2560	0.9211	512	0.9612
FOWFS-GA		2560	0.9212	1005	0.9579
FOWFS-PSO		2560	0.9245	905	0.9688
FOWFS-AJS		2560	0.9311	899	0.9779

**Table 8:** Recognition performance with respect to CNNs’ pre-trained models, CFS, and feature fusion configurations for HAM 10000 dataset.

Classifiers	Performance Measures	CNN Pre-Trained Models			CFS	Feature Fusion Configurations						
		VGG16	EfficientNet B0	ResNet50		AWFS	MOWFS-GA	MOWFS-PSO	MOWFS-AJS	FOWFS-GA	FOWFS-PSO	FOWFS-AJS
DT	Accuracy	0.9302	0.9321	0.9315	0.9412	0.9410	0.9411	0.9412	0.9424	0.9312	0.9412	0.9422
	Precision	0.9100	0.9112	0.9187	0.9189	0.9128	0.9254	0.9288	0.9321	0.9124	0.9311	0.9318
	Sensitivity	0.9125	0.9144	0.9128	0.9214	0.9311	0.9301	0.9299	0.9298	0.9388	0.9258	0.9301
	F1-Score	0.9115	0.9132	0.9120	0.9199	0.9205	0.9289	0.9289	0.9298	0.9298	0.9299	0.9304
NB	Accuracy	0.9308	0.9302	0.9311	0.9332	0.9411	0.9421	0.9422	0.9428	0.9398	0.9438	0.9448
	Precision	0.9104	0.9106	0.9111	0.9154	0.9128	0.9118	0.9187	0.9144	0.9218	0.9217	0.9288
	Sensitivity	0.9114	0.9114	0.9125	0.9128	0.9177	0.9178	0.9188	0.9198	0.9189	0.9200	0.9202
	F1-Score	0.9108	0.9110	0.9121	0.142	0.9135	0.9158	0.9187	0.9158	0.9199	0.9211	0.9245
MLP	Accuracy	0.9302	0.93	0.9342	0.9369	0.9550	0.9552	0.9558	0.9561	0.9349	0.9549	0.9569
	Precision	0.9114	0.9115	0.9105	0.9200	0.9189	0.9344	0.9341	0.9358	0.9219	0.9347	0.9382
	Sensitivity	0.9148	0.9198	0.9200	0.9258	0.9301	0.9289	0.9299	0.9351	0.9374	0.9387	0.9403
	F1-Score	0.9151	0.9144	0.9184	0.9235	0.9215	0.9288	0.9306	0.9352	0.9254	0.9355	0.9389
SVM	Accuracy	0.9412	0.9341	0.9416	0.9477	0.9599	0.9611	0.9712	0.9612	0.9479	0.9679	0.9779
	Precision	0.9204	0.9205	0.9345	0.9200	0.9301	0.9301	0.9289	0.9447	0.9321	0.9498	0.9524
	Sensitivity	0.9200	0.9236	0.9124	0.9258	0.9256	0.9306	0.9401	0.9400	0.9389	0.9498	0.9499
	F1-Score	0.9200	0.216	0.9205	0.9250	0.9289	0.9302	0.325	0.9411	0.9322	0.9497	0.9510

**Table 9:** Recognition performance with respect to CNN’s pre-trained models, CFS, and feature fusion configurations for BCN 20000 dataset.

Classifiers	Performance Measures	CNN Pre-Trained Models			CFS	Feature Fusion Configurations						
		VGG16	EfficientNet B0	ResNet50		AWFS	MOWFS-GA	MOWFS-PSO	MOWFS-AJS	FOWFS-GA	FOWFS-PSO	FOWFS-AJS
DT	Accuracy	0.9402	0.9461	0.9415	0.9512	0.9610	0.9611	0.9612	0.9624	0.9512	0.9612	0.9622
	Precision	0.9348	0.9311	0.9321	0.9348	0.9410	0.9422	0.9148	0.9522	0.9432	0.9498	0.9509
	Sensitivity	0.9218	0.9302	0.9109	0.9358	0.9389	0.9401	0.9487	0.9451	0.9422	0.9502	0.9511
	F1-Score	0.9225	0.9310	0.9215	0.9250	0.9399	0.9410	0.9255	0.9458	0.9425	0.9500	0.9510
NB	Accuracy	0.9421	0.9402	0.9451	0.9532	0.9611	0.9621	0.9622	0.9628	0.9598	0.9638	0.9648
	Precision	0.9215	0.9244	0.9348	0.9324	0.9422	0.9502	0.9248	0.9100	0.9458	0.9519	0.9588
	Sensitivity	0.9257	0.9301	0.9108	0.9458	0.9109	0.9002	0.9315	0.9487	0.9518	0.9505	0.9522
	F1-Score	0.9222	0.9241	0.9210	0.9344	0.324	0.9542	0.9268	0.9214	0.9461	0.9510	0.9544
MLP	Accuracy	0.9502	0.9538	0.9542	0.9569	0.9650	0.9652	0.9658	0.9661	0.9549	0.9649	0.9669
	Precision	0.9325	0.9328	0.9212	0.9318	0.9458	0.9428	0.9478	0.9488	0.9498	0.9500	0.9582
	Sensitivity	0.9388	0.9399	0.9458	0.9222	0.9331	0.9411	0.9501	0.9499	0.9502	0.9312	0.9401
	F1-Score	0.9341	0.9349	0.9332	0.9288	0.9339	0.9412	0.9481	0.9492	0.9499	0.9514	0.9554
SVM	Accuracy	0.9522	0.9541	0.9546	0.9572	0.9599	0.9611	0.9712	0.9612	0.9579	0.9679	0.9779
	Precision	0.9401	0.9388	0.9406	0.9399	0.9401	0.9402	0.9500	0.9502	0.9501	0.9515	0.9624
	Sensitivity	0.9358	0.9412	0.9402	0.9388	0.9385	0.9366	0.9488	0.9412	0.9499	0.9489	0.9539
	F1-Score	0.9366	0.9391	0.9404	0.9389	0.9390	0.9389	0.9489	0.9488	0.9488	0.9490	0.9568

## Data Availability

Data will be made available on request to the first author.
